# Reprogramming the tumour microenvironment by radiotherapy: implications for radiotherapy and immunotherapy combinations

**DOI:** 10.1186/s13014-020-01678-1

**Published:** 2020-11-04

**Authors:** Madyson Colton, Eleanor J. Cheadle, Jamie Honeychurch, Tim M. Illidge

**Affiliations:** 1 Division of Cancer Sciences, Manchester Academic Health Science Centre, NIHR Biomedical Research Centre, University of Manchester, Manchester, UK; 2 The Christie NHS Foundation Trust, Manchester, UK

**Keywords:** Radiotherapy, Radiation therapy, Immunotherapy, Immune checkpoints, Immuno-oncology agents

## Abstract

Radiotherapy (RT) is a highly effective anti-cancer therapy delivered to around 50–60% of patients. It is part of therapy for around 40% of cancer patients who are cured of their disease. Until recently, the focus of this anti-tumour efficacy has been on the direct tumour cytotoxicity and RT-induced DNA damage. Recently, the immunomodulatory effects of RT on the tumour microenvironment have increasingly been recognized. There is now intense interest in potentially using RT to induce an anti-tumour immune response, which has led to rethinking into how the efficacy of RT could be further enhanced. Following the breakthrough of immune check point inhibitors (ICIs), a new era of immuno-oncology (IO) agents has emerged and established immunotherapy as a routine part of cancer treatment. Despite ICI improving outcomes in many cancer types, overall durable responses occur in only a minority of patients. The immunostimulatory effects of RT make combinations with ICI attractive to potentially amplify anti-tumour immunity resulting in increased tumour responses and improved outcomes. In contrast, tumours with profoundly immunosuppressive tumour microenvironments, dominated by myeloid-derived cell populations, remain a greater clinical challenge and RT may potentially further enhance the immunosuppression. To harness the full potential of RT and IO agent combinations, further insights are required to enhance our understanding of the role these immunosuppressive myeloid populations play, how RT influences these populations and how they may be therapeutically manipulated in combination with RT to improve outcomes further. These are exciting times with increasing numbers of IO targets being discovered and IO agents undergoing clinical evaluation. Multidisciplinary research collaborations will be required to establish the optimal parameters for delivering RT (target volume, dose and fractionation) in combination with IO agents, including scheduling to achieve maximal therapeutic efficacy.

## Background

Radiotherapy (RT) is a highly effective anti-cancer therapy known to induce direct DNA damage to tumour cells. More recently, the immunomodulatory effects of RT on the tumour microenvironment (TME) has encouraged investigations into how RT efficacy might be enhanced. RT is able to induce a local anti-tumour immune response, potentially leading to systemic anti-tumour immunity and contributing to tumour regression outside of the local radiation field, termed the “abscopal effect” [1]. Though reports of abscopal effects date back to 1953, systemic responses remain an extremely rare clinical occurrence [2]. Therefore, the research focus has been on understanding the mechanisms of RT induced anti-tumour immunity and potentially manipulating this further with therapeutic immuno-oncology (IO) agents to increase the frequency of systemic responses. There has been a surge of interest in IO agents following the clinical success of Ipilimumab in the treatment of metastatic melanoma [3]. Ipilimumab is a monoclonal antibody (mAb) that targets Cytotoxic T Lymphocyte Associated Protein 4 (CTLA-4), inhibiting its actions as a suppressive immune checkpoint and thereby facilitating an anti-tumour immune response. This success has led to the development of other immune checkpoint inhibitors (ICIs), with durable remissions observed using mAbs against Programmed Cell Death Protein/Ligand 1 (anti-PD1/PD-L1) in numerous disease groups and improved survival in metastatic disease. Despite ICIs being recognized as breakthrough therapies, only the minority of cancer patients respond to such treatment [4–6].

The immunomodulatory potential of both RT and ICIs has provided rationale for combining RT and IO agents to further improve overall response rates and the duration of responses. Rare abscopal effects are now increasingly reported as case reports in patients receiving combined treatment, but they are still the rare minority and further prospective clinical trials are required [7–11]. While many pre-clinical studies investigating RT and IO combinations demonstrate proof of principle with long-term tumour control in murine tumour models, translating this into clear clinical benefit has proved challenging [12–15]. In a study of metastatic lung cancer patients, combining anti-CTLA4 mAb with RT induced systemic responses where anti-CTLA4 alone had failed; however, disease control was only achieved in 31% (12/39) of patients with only two complete responses [16]. Further, in the TONIC trial, no benefit was observed from combining single-site RT with anti-PD1 [17].

**Fig. 1:**
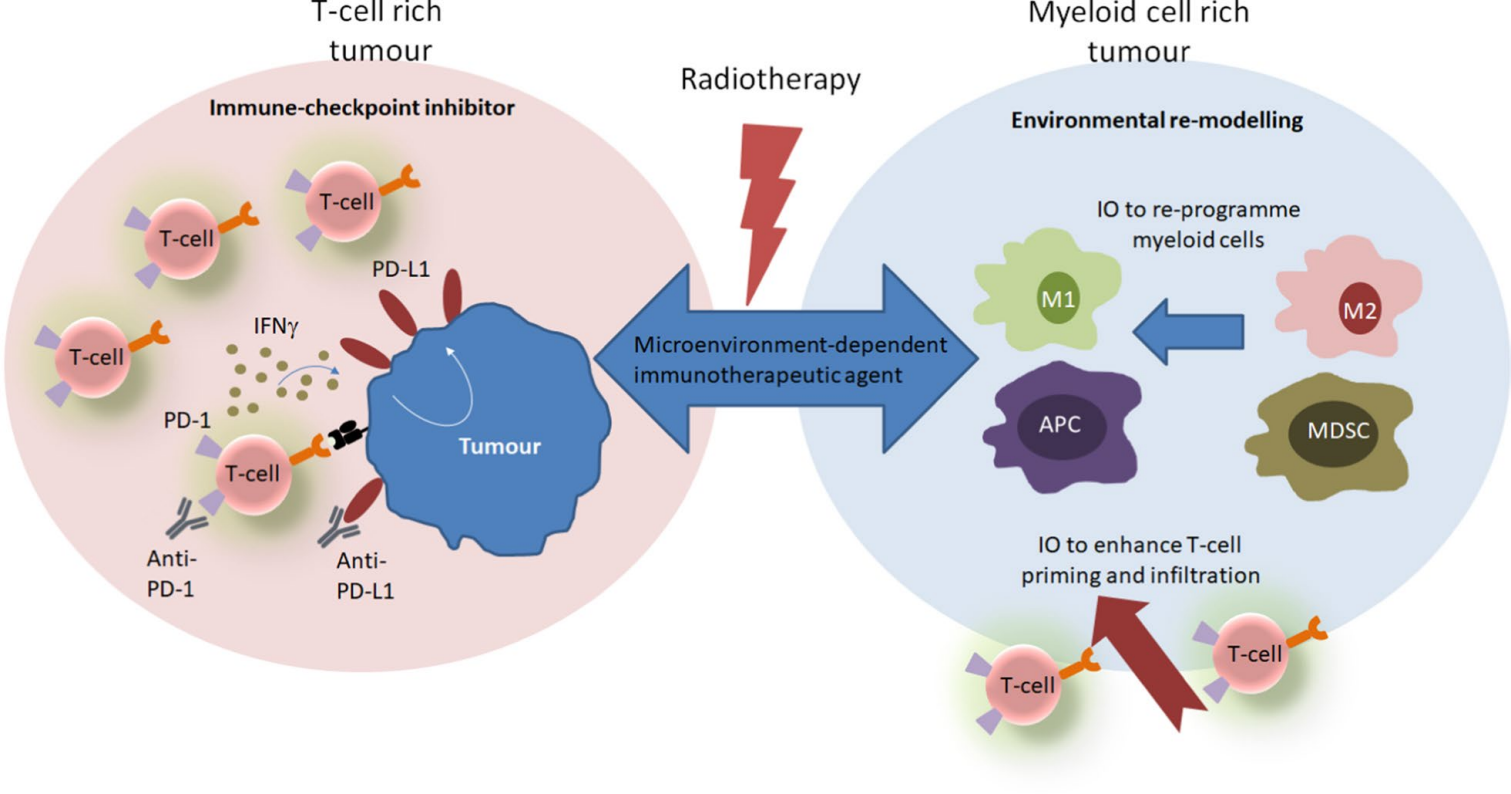
The choice of therapeutic agent in combination with radiotherapy may depend on the TME. Radiotherapy can drive the expansion and activation of T-cells in tumours with a T-cell rich TME. This leads to upregulation of PD-L1 and these tumours respond to ICIs. Conversely, tumours with a myeloid rich TME do not respond to ICI, RT drives further expansion and suppressive myeloid cells and may need to be combined with IO agents which re-programme myeloid cells and/or enhance T-cell priming and infiltration. *ICI* immune checkpoint inhibitor, *IO* immuno-oncology, *M2* M2-polarised macrophage, *mAb* monoclonal antibody, *MDSC* myeloid-derived suppressor cell, *RT* radiotherapy, *TME* tumour microenvironment

The immune contexture of the TME is now understood to be a significant predictive biomarker of response to immunotherapies. Tumours with an abundance of infiltrating T-cells appear to be most likely to respond to ICI, whereas tumours with an abundance of immunosuppressive myeloid cells and few infiltrating T-cells fail to exhibit a durable response [18–21]. RT may enhance the ability of T-cell rich tumours to respond to ICI but to compound matters further, RT may also modulate the TME to support myeloid populations [22]. These discoveries have encouraged investigations into stimulatory IO agents which induce T-cell infiltration and activation, or which reprogram myeloid populations in tumours where RT drives immunosuppression (Fig. 1) [12, 23].

This review will therefore describe our current understanding of the complex interaction of RT with the TME, how IO agents may modulate this and the challenges of translating combined therapies into the clinic.

## The effects of RT on the induction of systemic immunity

### T-cell priming and antigen-specific immune response

The therapeutic success of RT has been shown to be dependent on effector T-cells and their ability to respond to tumour antigens [24]. Irradiation triggers immunogenic cell death (ICD) by inducing DNA damage and the subsequent release of damage-associated molecular patterns (DAMPs) from tumour cells, turning tumour cells into an “in situ vaccine” [25]. These effects promote dendritic cell (DC) antigen presentation, and the differentiation of naïve T-cells towards an effector phenotype.

Irradiated cells release DAMP signals which enhance the function of DCs. The release of adenosine triphosphate recruits DCs to the tumour. Calreticulin is translocated to the surface of dying cells where it is recognized by DCs, promoting phagocytosis. The passive release of HMGB1 enables DCs to efficiently process and cross-present antigens through toll-like receptor (TLR) 4 dependent signaling, inducing an effective T-cell mediated immune response [26–28]. Therefore, through stimulating DCs, RT is an effective adjuvant for immunotherapies.

Depletion studies in murine lymphoma models indicate that DCs—not B cells or macrophages—are indeed the major antigen presenting cell (APC) required for durable anti-tumour immunity, demonstrated when RT is combined with stimulatory CD40 mAb [29]. CD40 agonists are known to enhance DC function through increased surface expression of major histocompatibility complex (MHC) molecules and the production of pro-inflammatory cytokines [23]. Exploring novel strategies to augment DC function may improve therapeutic outcomes post-RT.

The presence of cytosolic DNA and micronuclei in irradiated tumour cells also activates cGas/STING signalling pathways which stimulate downstream production of immunogenic type I interferons (IFN), responsible for the maturation of DCs amongst other immunostimulatory events [30–34]. This pathway also activates CD8+ T-cells and has been shown to potentiate the effects of PD-L1 blockade [30, 35]. The cGas/STING cascade is negatively regulated by protective DNA damage response (DDR) pathways. Targeting DDR pathways via PARP or CHK1 inhibitors significantly increases surface expression of PD-L1 and augments cytotoxic T-cell infiltration in in vivo models of small cell lung carcinoma [36]. The PARP inhibitor Olaparib is therefore undergoing clinical trial evaluation in combination with anti-PD1 mAbs to assess clinical efficacy [31, 37]. Enhancing the effects of RT-induced DNA damage through DDR inhibitors is a logical approach to improving tumour response and is currently being extensively investigated; the addition of an ICI such as anti-PD1 to overcome tumour-induced immunosuppression is an exciting prospective approach [38].

**Fig. 2:**
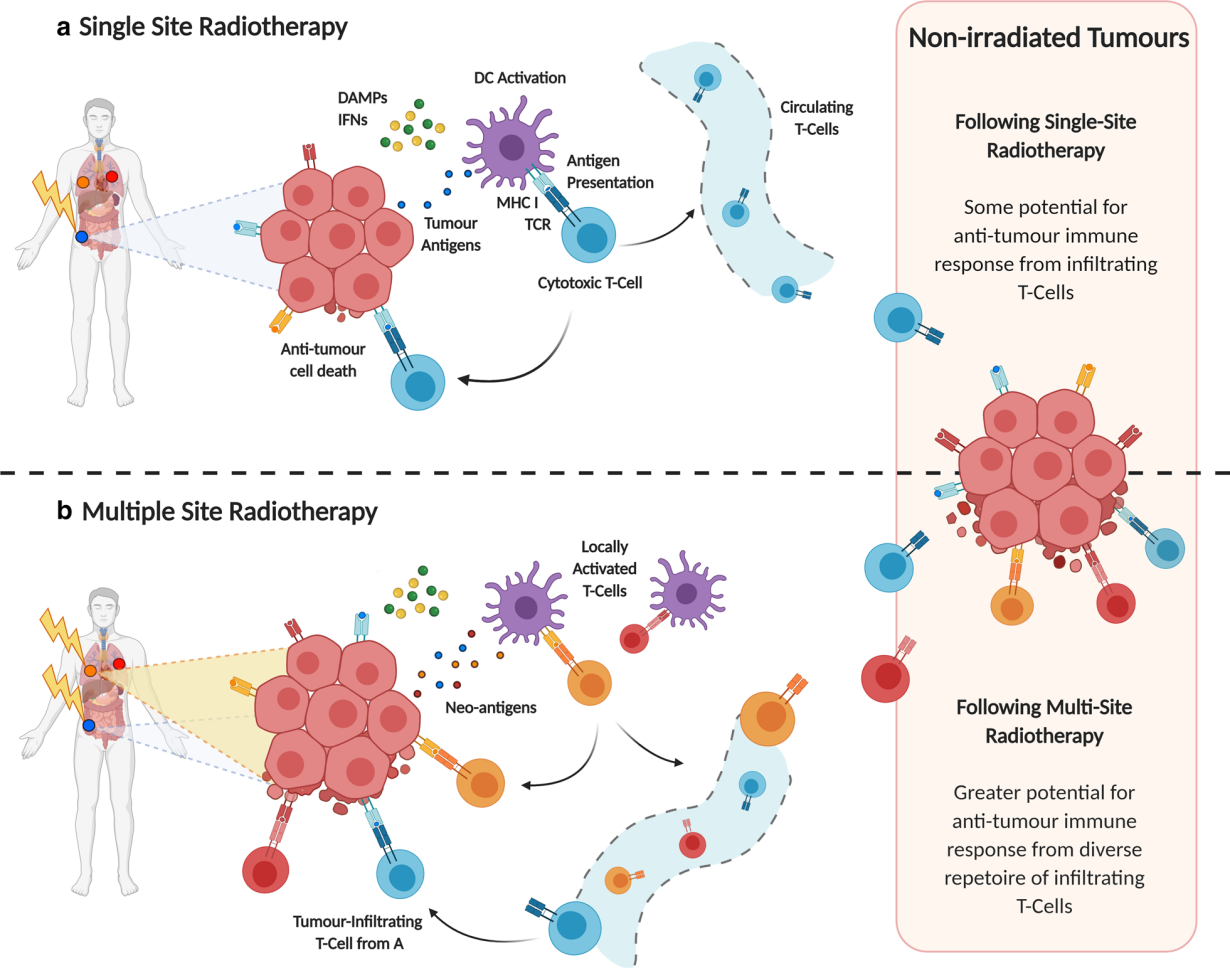
Radiotherapy induces a local anti-tumour immune response (**a**). Radiotherapy delivered to multiple sites may augment systemic responses (**b**). RT-induced immunogenic cell death stimulates the release of DAMPs and type 1 IFNs, which enhance antigen uptake and presentation by DCs. DCs present tumour antigens to T cell receptors, priming naïve T-cells to an effector phenotype. These T-cells migrate into the local tumour to exert their cytotoxic effects, or re-enter the circulation and migrate to distal, non-irradiated tumour sites (Panel A). At distal tumour sites, DCs may also activate T-cells against novel tumour antigens. RT delivered to multiple sites may therefore increase the quantity and diversity of migrating T-cells, enhancing the potential for systemic immune responses at non-irradiated sites (**b**). *DAMPs* damage-associated molecular patterns, *DC* dendritic cell, *IFN* interferon, *MHC I* major histocompatibility complex class I, *RT* radiotherapy, *TCR* T-cell receptor

RT has also been described to activate mTOR signalling as part of DDR pathways [39, 40]. This may lead to an increase in peptide presentation by tumours and recognition by effector T-cells [40]. Upon activation, T-cells produce IFNγ which increases antigen spread and further enhances MHC expression on tumour cells, augmenting immune recognition [41]. When cytotoxic CD8+ T-cells induce tumour cell death, they release new tumour antigens which further strengthens the immune response. This has led to the concept of RT-induced “in situ anti-tumour vaccination”, whereby the enhanced immunogenicity of the irradiated tumour might augment systemic responses, as shown in Fig. 2, panel A [42–44].

### T-cell trafficking: recruitment and migration

Both resident and infiltrating T-cell populations appear to be required for effective control of irradiated tumours and distal tumour sites following RT and anti-PD1 combinations [45]. Local RT may initially induce cell death in radiosensitive tumour-residing T lymphocytes, but it may also stimulate T-cell tumour infiltration.

T-cell recruitment to the TME is mediated by adaptive and innate immune responses induced by RT. Once matured, DCs migrate to tumour draining lymph nodes (LNs) where they prime naïve T-cells towards an effector phenotype. These effector T-cells, along with CD4 + T helper cells, are trafficked to the tumour site via chemokine gradients [46, 47]. RT damage also induces increased expression of adhesion molecules ICAM and VCAM1 on tumour and endothelial cells, which attracts effector T-cells. Blocking infiltrating T-cells from binding to these adhesion molecules impedes T-cell mediated tumour rejection, which highlights the significance of leukocyte recruitment in establishing anti-tumour immunity [48].

Work from our own laboratory investigating T-cell receptor clonality has suggested that following RT and anti-PD1 combinations, the immune response in the local TME is dominated by polyclonal expansion of pre-existing T-cell clones [45]. The eradication of secondary tumours outside of the local radiation field was only observed in mice receiving the RT and anti-PD1 combination therapy. It is unclear whether this systemic response was dependent on the migration of pre-existing T-cells clones or de novo activation of T-cells at the secondary site, but both resident and infiltrating T-cells were required for primary response. On-going research aims to elucidate the mechanism of action of T-cell trafficking to better understand how combination approaches succeed in targeting distal metastases.

Pre-clinical studies of RT-anti-PD1 combinations suggest that irradiating draining LNs impacts T-cell infiltration into the primary tumour, modifies intra-tumoural chemokine expression and reduces overall survival [49]. Since the lymphatic system plays a crucial role in the migration of DCs and T-cells, it is likely that irradiating LNs impedes cell migration away from the primary tumour towards distal sites. Understanding the impact of LN irradiation on systemic immunity may shape clinical decisions for elective nodal irradiation.

Further studies are also needed to clarify the significance of T-cell trafficking in phenotypically immunosuppressive tumours, such as prostate cancers. These may benefit from IO agents which stimulate leukocyte trafficking, such as anti-CD40. The administration of agonistic CD40 alone or in combination with RT leads to a significant increase in CD8+ and CD4 + T-cells infiltrating the tumour, and the combined approach may successfully induce T-cell dependent immunity [50–52].

The infiltration of T-cells with a memory phenotype (CD8+ , CD103 +) into the TME correlates with improved overall survival in lung and ovarian tumours [53, 54]. Resident memory T-cells (Trm) are a recently identified subset of T-cells which reside in the tissue without recirculating and are linked to enhanced tumour control. In mouse models of melanoma, anti-PD1 treatment enhances infiltration of plastic circulating memory T-cells, which give rise to Trm cells [55]. Both cell subsets express PD1 and the presence of either memory T-cell is sufficient to induce an immune response, though Trm cells do so with a stronger efficacy. In another study, combining RT-anti-PD1 with an additional mAb against the macrophage-specific phagocytic receptor Mer-TK delayed abscopal tumour growth through the retention of Trm cells in the secondary TME [56]. Further studies are needed to better understand the impact of RT on Trm populations and the significance of these cells in inducing abscopal responses.

## The impact of myeloid cell populations on RT and immunotherapy combinations

Tumours with a high proportion of myeloid and other suppressive effector cells within the TME can hinder anti-tumour immune responses. These tumours are radioresistant and lack clinical responsiveness to ICI [18, 57–60]. Despite the immunostimulatory effects discussed above, RT can encourage an immunosuppressive TME through the recruitment of myeloid-derived suppressor cells (MDSC) populations and the repolarization of macrophages into an M2 phenotype [61].

Immediately following RT, there is a relative increase in MDSCs, tumour associated macrophages (TAMs) and regulatory T-cells (Tregs) within the TME, as these appear to be more radioresistant than T lymphocytes [62]. MDSCs and TAMs drive tumour growth and angiogenesis whilst negatively regulating T-cells [58, 63, 64]. Tregs contribute to immunosuppression partially via CTLA4 checkpoint signaling which inhibits cytotoxic T-cell activation [65, 66]. Irradiated tumour cells release oxygen and nitrogen radicals, which encourage the polarization of macrophages from an inflammatory M1 phenotype into a tumour-supporting M2 phenotype. These M2 TAMs secrete cytokines and matrix metalloproteinases which assist tumour immune evasion [67]. Repopulation of the TME with T-cells following RT will therefore be vital for anti-tumour immune responses.

RT stimulates the recruitment of suppressive cells to the TME by increasing the expression of inflammatory chemokines such as CXCL5 and CXCL2; these appear important in recruiting MDSCs and Tregs via STING-activated CCR2 pathways, as well as stimulating the production of TGFβ [68, 69]. TGFβ is well known for its role in supporting tumour progression and has recently been shown to impede anti-tumour immunity post-RT [70, 71]. Within the TME, TGFβ suppresses the effector functions of T-cells and natural killer cells, and inhibits DC maturation. TGFβ also promotes M2 macrophage polarity and favours the conversion of CD4 + T-cells into immunosuppressive Tregs. In murine models of colorectal cancer (CRC), mice receiving a TGFβ inhibitor were resistant to metastatic formation [72]. However, utilizing TGFβ inhibitors to improve RT efficacy has not consistently induced tumour control due to the complex role played by this cytokine as both a tumour promotor and tumour suppressor [68, 73]. RT also stimulates production of chemokines CCL2 and CCL5, which recruit inflammatory monocytes that differentiate into immunosuppressive TAMs in the TME. Dual CCL2/CCL5 antagonists which target these monocytic populations improve RT efficacy and reduce tumour metastases in poorly immunogenic breast and pancreatic tumour models, highlighting the significance of myeloid populations [74–76].

MDSC have been shown to contribute to patient resistance to ICI [77–79]. RT and anti-PD1 combinations fail to induce long-term tumour clearance in tumours with high populations of myeloid cells residing in the TME [57]. Circulating myeloid populations also appear to influence therapeutic response, as high peripheral levels of monocytic MDSC are associated with greater tumour burden, lower numbers of antigen-specific T-cells and resistance to ICIs [19]. In the treatment of melanoma with Ipilimumab, non-responders demonstrated significantly higher proportions of MDSC, neutrophils and monocytes in the TME [77]. MDSC populations in the TME have also been negatively correlated with the efficacy of DC-based immunotherapies in combination with RT [58, 80].

The utilization and development of therapeutic agents which manipulate or reprogram myeloid populations provide another exciting opportunity to improve clinical responses to RT-IO combinations. Stimulatory IO agents employed to activate anti-tumour T-cell responses can also reprogram suppressive cell populations. CD40 agonists manipulate macrophages to acquire an M1 phenotype, thereby upregulating pathways associated with effector T-cell priming [81]. Macrophage-specific MerTK is also a therapeutic target of interest, with improved survival rates seen with inhibitory mAbs combined with RT-anti-PD1 in lung adenocarcinomas, and tumour regression when combined with RT and TGFβ blockade in poorly immunogenic tumour models [56, 82]. TLR signalling is central to several stages of T-cell activation, making TLRs an attractive therapeutic target [83, 84]; TLR agonists may also repolarise macrophages to an M1 phenotype and convert MDSCs into APCs, stimulating T-cell responses [85–87]. In preclinical studies, RT potentiates the effects of TLR7/8 agonists, inducing durable anti-tumour immune responses and reduced metastases in several disease subsets [12, 88–90].

Targeting TGFβ represents another potential strategy to enhance combination therapies. TGFβ inhibitors in combination with anti-PD1 mAbs demonstrated CD8+ T-cell infiltration and improved tumour control in a subset of urothelial cancer patients and poorly immunogenic breast cancer models [71, 91]. Promising results were also seen in metastatic CRC models in which TGFβ inhibition sensitized previously unresponsive tumours to anti-PD1 [92]. RT-induced TGFβ was shown to inhibit abscopal responses even in combination with anti-PD-1 and anti-CD137 mAbs; this was overcome by TGFβ blockade [93]. TGFβ blockade has also demonstrated promising results in combination with an agonistic OX40 mAb in metastatic breast cancer, where there was a synergistic T-cell dependent response [94]. Clinical trials are under development to study triple-combinations of TGFβ inhibitors, RT and ICIs in non-small cell lung carcinoma (NSCLC), hepatocellular carcinoma and pancreatic tumours [95, 96].

Phosphodiesterase-5 (PDE5) inhibitors are currently approved for non-malignant conditions and have been shown to reduce the immunosuppressive capacity of MDSCs in murine tumour models. PDE5 inhibitors increase T-cell infiltration and activation through Arg-1 and NOS down-regulation resulting in improved efficacy of adoptive T-cell therapies [97]. In clinical trials of HNSCC and metastatic melanoma, the PDE5 inhibitor Tadalafil improved clinical outcomes and augmented immune responses by reducing MDSC function [98]. This provides rational for trialing PDE5 inhibitors in RT and immunotherapy combinations. There are currently several clinical trials underway investigating the therapeutic manipulation of myeloid populations in conjunction with RT and ICI [18]. However, further studies are needed to fully elucidate the impact of these populations on the efficacy of treatments and determine whether their reprogramming may overcome resistance to RT-ICI combinations.

Hypoxia has long been established as a significant factor in radioresistance [99]. More recently, the effect of oxygen-deficient TMEs on immunosuppressive cell populations has emerged as potentially important [61]. RT is able to support hypoxic environments by disrupting tumour vasculature and the generation of reactive oxygen species [100–103]. Hypoxic tumours produce potent T-cell suppressor adenosine, recruit MDSCs and TAMs via CSF1 signalling, and further support immunosuppression via TGFβ signalling [61, 104]. The expression of hypoxia-inducible factor-1 (HIF-1) stimulates secretion of stromal-derived factor-1 which further recruits MDSCs via CXCR4 binding [61]. Through HIF-1, hypoxia also upregulates PD-L1 expression on MDSCs and tumour cells. Patients with hypoxic tumours may therefore benefit from anti-PD1 mAbs [104]. However, disrupted vasculature in hypoxic tumours may hinder the delivery of IO agents to the tumour site. The addition of a therapeutic agent to re-oxygenate tumours, such as nitrous oxide, is therefore being considered to increase the frequency of responders to combination therapies [105, 106].

## Clinical considerations for the delivery of RT and immunotherapy combinations

Due to the complex immunological interplay between RT and IO agents (Table 1), there are many uncertainties regarding how the delivery of RT may impact immunotherapy efficacy. Establishing the optimal RT dose, fractionation and target volume along with the optimal scheduling of IO agents are just some factors required for successful clinical translation.

**Table 1:** Summary of immuno-oncology agents that could be combined with radiotherapy to improve patient outcomes

Agent	Rationale for combining with radiotherapy	References
CTLA-4 Inhibitor	Immune checkpoint inhibitor. Combination with RT has induced responses in patients where anti-CTLA4 alone had failed. Systemic responses have been observed in patients receiving RT + anti-CTLA4	[3, 7, 8, 16]
PD-1/PD-L1 Inhibitor	Immune checkpoint inhibitor. Systemic responses have been observed when combined with RT. Increased progression free survival and overall survival observed in patients with NSCLC who received RT + anti-PD1	[9, 117, 118]
CD40 Agonist	Enhances DC function, stimulates T-cell trafficking, and activates M1 polarized macrophages, so may overcome immunosuppression. Successful anti-tumour immune responses observed in mice receiving RT + CD40	[29, 50, 52, 81]
TLR Agonist	Activates T-cells, blocks immunosuppressive effects of MDSCs and tumour associated macrophages. May convert MDSC into immunostimulatory antigen presenting cells	[83–90]
CCL2/5 Inhibitor	Prevents monocyte recruitment to the tumour microenvironment and improves responses to RT in pre-clinical studies	[74–76]
Mer-TK Inhibitor	Inhibits tumour associated macrophages. Tumour regression observed when combined with RT. Induced responses in ‘cold’ tumours with the addition of RT and a TGFβ inhibitor. Delayed metastasis and improved survival when combined with anti-PD1 and RT in pre-clinical studies	[56, 82]
PARP Inhibitor	Inhibits tumour damage response pathways. Increases T-cell infiltration and increases PD-L1 expression, so could be combined with anti-PD1 and RT	[35–38]
TGFβ Inhibitor	Inhibits immunosuppressive effects of TGFβ. Enhances T-cell infiltration in combination with anti-PD1. Combination with RT and anti-PD1 induced greater responses compared to anti-PD1 alone	[72, 73, 93, 95]
PDE5 Inhibitor	Increases T cell infiltration and activation by reducing MDSC function. Improved outcomes observed in patients with metastatic melanoma	[97, 98]

The dose and number of fractions of RT will likely play a critical role in the immunomodulation of the TME. High-doses of 12–16 Gy delivered in a single fraction induce protective DDR pathways within the tumour which hinder T-cell response, whereas lower doses have been shown to optimally induce the production of IFNβ required for DC activation [33]. It is currently unclear whether single high-doses or fractionated low-doses would better complement ICIs., High-dose RT (12 or 20 Gy) has been shown to increase PD-L1 expression on tumour cells, where anti-PD1 treatment can induce successful tumour control [13, 14, 107, 108]. Conversely, lower doses given in fractions (18 × 2 Gy or 5 × 2 Gy) have also been shown to increase PD-L1 expression and may result in earlier expression, suggesting that further studies into optimal RT doses are required [109, 110]. Though higher doses may induce cell death in lymphocytes, it is likely that different T-cell populations exhibit differing sensitivities to RT, as Trm population have been demonstrated to increase in proportion following local irradiation of solid tumours [111, 112]. IFNγ, which is found at greater levels within the irradiated tumour than at secondary sites, can also mediated T-cell survival post RT, suggesting targeting multiple tumour sites could increase the efficacy of immune responses [111, 113, 114].

Currently the majority of clinical trials investigating RT-IO combinations employ single site irradiation, which may not be optimal. In the situation of multiple metastases, multi-site irradiation may improve therapeutic outcomes by reducing disease burden and also by increasing RT-induced immune stimulation, as shown in Fig. 2, panel B [115, 116]. In this situation, RT delivery to a single tumour may not liberate enough tumour antigens to generate a sufficiently robust systemic anti-tumour immune response. Increasing RT target volume to reduce disease burden and increase immune infiltration may therefore enhance the efficacy of IO agents. However, consideration must also be given to the potential increase in patient toxicity associated with large volume fields and multiple sites of RT, as well as the potential for RT to induce cell death in radiosensitive immune cells within the tumour and in local LNs [49]. Further research is required to address these important questions.

Scheduling of the IO agent relative to the delivery of RT is also likely to affect the generation of systemic immune responses. The optimal schedule is likely to depend on disease group, tumour site and the RT-IO combination employed. Research from our laboratory in murine models of CRC evaluated three regimes of RT with anti-PD1: concurrent delivery at the start of RT cycle; concurrent delivery at the end of RT cycle; sequential delivery 7 days after RT completion. Acquired resistance to radiotherapy was overcome by concurrent delivery of anti-PD1 with effective anti-tumour immunity and tumour control; sequential delivery was less effective [13]. However, the recently published PACIFIC trial of NSCLC patients demonstrated improved progression-free survival with sequential anti-PD1 delivery, with the greatest benefit seen in patients who began anti-PD1 in the shortest timeframes to completing RT [117]. This emerging data highlights the importance of scheduling RT with IO agents and the requirement for further study across different tumour types.

Further investigations are also needed to identify biomarkers which inform on RT-induced immunological changes, which will guide decision making in the clinic. Predictive biomarkers to identify the most appropriate IO agent for each patient would increase the frequency of responders. PD-L1 expression has emerged as a potential prognostic biomarker correlated with improved survival after RT, though its value as a predictive biomarker for RT-IO agent combinations remains to be seen [118–120]. There has been some success using tumour immune infiltrates—the tumour ‘immunoscore’—to predict RT outcomes, though again further studies should investigate its predictive value for combination therapies [121–124]. Due to the complex interplay between RT and the anti-tumour immune response, surveillance of patients throughout treatment using dynamic biomarkers would allow for real-time decision making to improve treatment efficacy and prevent toxicities. Therefore, there is an urgent need for studies to monitor RT-induced changes to the immune microenvironment throughout treatment and across tumour types, and for clinical trials to include predictive biomarker discovery in their study outcomes.

## Conclusion

The ability of RT to reprogram the TME has complex local and systemic consequences. The immunostimulatory effects of RT make combinations with IO agents attractive to amplify effective anti-tumour immunity and improve outcomes. In contrast, immunosuppressive TMEs dominated by MDSC populations remain a greater clinical challenge, as RT may potentially enhance immunosuppression. To harness the full potential of RT-IO agent combinations, further insights are required to understand the role of these immunosuppressive myeloid populations, how RT influences them and the optimal ways to therapeutically manipulate them to improve clinical outcomes. These are exciting times with increasing numbers of IO targets being discovered and undergoing clinical evaluation [125–127]. The experience with ICI informs us that it is unlikely that a single IO agent will be sufficient to induce durable anti-tumour immunity in all patients, and so combination approaches will be required. Using multiple agents will create further challenges related to toxicity and adverse effects. Multidisciplinary research collaborations will be required to establish the optimal target volume, dose and fractionation to deliver RT in combination with IO agents to achieve maximal therapeutic efficacy. Discovering dynamic RT-related biomarkers will also be critical for translation. Prognostic and predictive immune biomarkers will enable clinicians to assess the immune microenvironment throughout RT, predict patient benefit from therapeutic agents and monitor their response, enabling patient treatment plans to be personalised.

## Data Availability

Not applicable.

## Abbreviations

APC: Antigen presenting cell
CRC: Colorectal cancer
CTLA4: Cytotoxic T lymphocyte associated protein 4
DAMP: Damage associated molecular patterns
DC: Dendritic cell
DDR: DNA damage response
HIF1: Hypoxia inducible factor 1
HMGB1: High mobility group box protein 1
HNSCC: Head and neck squamous cell carcinoma
ICAM: Intracellular adhesion molecule
ICD: Immunogenic cell death
ICI: Immune checkpoint inhibitor
IFN: Interferon
IO: Immuno-oncological
LN: Lymph node
mAb: Monoclonal antibody
MDSC: Myeloid derived suppressor cell
MHC: Major histocompatibility complex/class 1
mTOR: Mammalian target of rapamycin
NSCLC: Non small cell lung carcinoma
PARP: Poly (ADP-ribose) polymerase
PD-1/PD-L1: Programmed cell death protein/ligand 1
PDE-5: Phosphodiesterase-5
RT: Radiotherapy
STING: Stimulator of interferon genes
TAM: Tumour associated macrophages
TGFβ: Transforming growth factor beta
TLR: Toll-like receptor
TME: Tumour microenvironment
Tregs: Regulatory T-cells
Trm: Resident memory T-cells
VCAM: Vascular cell adhesion molecule
